# Antimicrobial Susceptibility of Canine and Feline Urinary Tract Infection Pathogens Isolated from Animals with Clinical Signs in European Veterinary Practices during the Period 2013–2018

**DOI:** 10.3390/antibiotics13060500

**Published:** 2024-05-28

**Authors:** Robin Temmerman, Helena Berlamont, Farid El Garch, Markus Rose, Shabbir Simjee, Sylvie Meschi, Anno de Jong

**Affiliations:** CEESA ComPath Study Group, 1050 Brussels, Belgium

**Keywords:** surveillance, antimicrobial resistance, urinary tract infections, companion animals

## Abstract

Bacterial urinary tract infections (UTIs) occur frequently in companion animals and are often treated with antibiotics. However, antimicrobial resistance can severely hamper treatment success. Therefore, antimicrobial susceptibility monitoring is key. UTI isolates were obtained from dogs and cats in two collection periods (ComPath II: 2013–2014 and ComPath III: 2017–2018) as part of CEESA’s ComPath programme. Susceptibility testing of the UTI isolates (2021 in total) was carried out at one central laboratory using agar and broth dilution methodology as recommended by the Clinical and Laboratory Standards Institute. *Escherichia coli* was the most frequently isolated bacterium in UTI in both dogs (46.9%, 43.1%) and cats (61.2%, 48.3%) across ComPath II and ComPath III, respectively. The percentage of resistance in *E. coli* was low (<10%) across both programmes in both dogs and cats except for trimethoprim-sulfamethoxazole (dogs ComPath III: 12.9%; cats ComPath II: 13.0%) and enrofloxacin (10.5%), marbofloxacin (11.4%), and doxycycline (98.8%) for dogs in ComPath III. Three (7.5%) of the 40 isolated *S. aureus* bacteria in total were MRSA and harboured *mecA*. The level of multidrug resistance (MDR) was generally low and ranged from 0.0% for feline coagulase-negative *Staphylococcus* spp. to 11.7% for canine *Proteus* spp., except for a peak of MDR observed in canine *Klebsiella* isolates from ComPath II (36.7%). Overall, antimicrobial resistance for most canine and feline UTI pathogens isolated during the ComPath II and ComPath III programmes was low (1–10%) to moderate (10–20%).

## 1. Introduction

Bacterial urinary tract infections (UTIs) are one of the most common morbidities in companion animals [1]. Incidences of UTIs in dogs and cats are estimated to be 14% and 3–19% (depending on the inclusion criteria of investigating studies), respectively, and constitute a major indication for antibiotic use in dogs and cats [2,3,4]. UTIs are frequently empirically treated with antibiotics, i.e., without determination of the pathogen species and antimicrobial susceptibility testing [5]. As always, antimicrobial use can lead to selection of resistance, calling for monitoring of antimicrobial susceptibility and guidance for choosing the most appropriate drug for treatment of UTIs. Since humans and pets are sharing living spaces, the potential for transmission of antimicrobial resistant bacteria or their resistance determinants from companion animals to humans and vice versa has been a public health concern for many years [1,6,7]. The World Health Organization (WHO) considers antimicrobial resistance to be an urgent global health threat and has worked out a global action plan on antimicrobial resistance in the context of a One Health approach [8].

The International Society for Companion Animal Infectious Diseases (ISCAID) first published guidelines for the diagnosis and management of bacterial UTIs in dogs and cats in 2011, and revised in 2019 [4,9]. These guidelines are based on available data from veterinary and human medicine, along with expert opinions, considering principles of infectious diseases, antimicrobial therapy and resistance, pharmacology, and internal medicine [4]. Given the frequency of UTIs, the commonalities between veterinary and human uropathogens, and the known issues with antimicrobial resistance in these pathogens, regular updates to data on antimicrobial resistance are needed for optimal guidance in treatment management of bacterial UTIs.

The CEESA (Centre Européen d’Etudes pour la Santé Animale) ComPath programmes are based on harmonized methods of sampling and bacterial isolation to establish pan-European collections of representative pathogens from diseased companion animals not recently exposed to antimicrobial treatment [10,11,12,13]. The ComPath programmes are currently the only international surveillance programmes monitoring companion animal UTI pathogen resistance on a European scale with large numbers of isolates [14]. UTI pathogen isolates in the present study were obtained in two study periods (ComPath II: 2013–2014 and ComPath III: 2017–2018), through which antimicrobial susceptibility data were collected for the entire collection in a single central laboratory to mitigate inter-laboratory variation [11,12,13]. The results on UTI pathogens of the ComPath I programme (2008–2010) have already been published [12].

## 2. Results

### 2.1. Isolated Bacteria

#### 2.1.1. General

A total number of 869 (606 in dogs, 263 in cats) and 1152 (773 in dogs, 379 in cats) isolates were recovered over the 2013–2014 and 2017–2018 collection periods, respectively (2021 isolates in total). The age of the dogs from which the isolates were collected ranged from 0 to 22 years old in ComPath II and from 0 to 20 years old in ComPath III, and the age of the cats ranged from 0 to 18 years old in ComPath II and from 0 to 22 years old in ComPath III. From the 606 dogs in ComPath II, 233 (38.4%) were male, 327 (54.0%) female, and 46 (7.6%) not defined. From the 263 cats in ComPath II, 114 (43.3%) were male, 133 (50.6%) female, and 16 (6.1%) not defined. From the 773 dogs in ComPath III, 261 (33.8%) were male, 474 (61.3%) female, and 38 (4.9%) not defined. From the 379 cats in ComPath III, 148 (39.0%) were male, 219 (57.8%) female, and 12 (3.2%) not defined.

**Figure 1:**
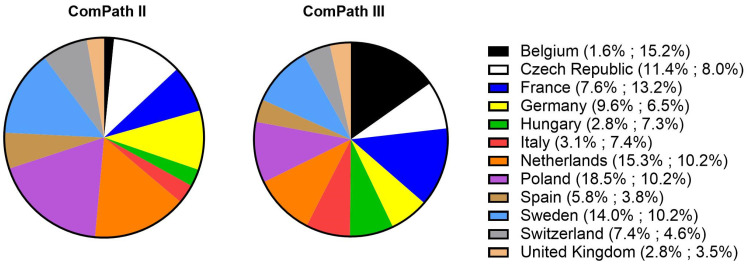
Relative proportion of isolated bacteria from each participating country in the two collection programmes (ComPath II (2013–2014) and ComPath III (2017–2018)).

Of the collected isolates during the ComPath II programmes (2013–2014), 1.8% originated from Belgium, 11.4% from the Czech Republic, 7.6% from France, 9.6% from Germany, 2.8% from Hungary, 3.1% from Italy, 15.3% from Netherlands, 18.5% from Poland, 5.8% from Spain, 14.0% from Sweden, 7.4% from Switzerland, and 2.8% from the UK. For the ComPath III programmes (2017–2018), the percentages were 15.2% (B), 8.0% (CZ), 13.2% (F), 6.5% (D), 7.3% (H), 7.4% (I), 10.2% (NL), 10.2% (P), 3.8% (E), 10.2% (SW), 4.6% (CH), and 3.5% (UK). The relative proportion of isolates from each country in the two collection programmes has been summarized in Figure 1.

**Figure 2:**
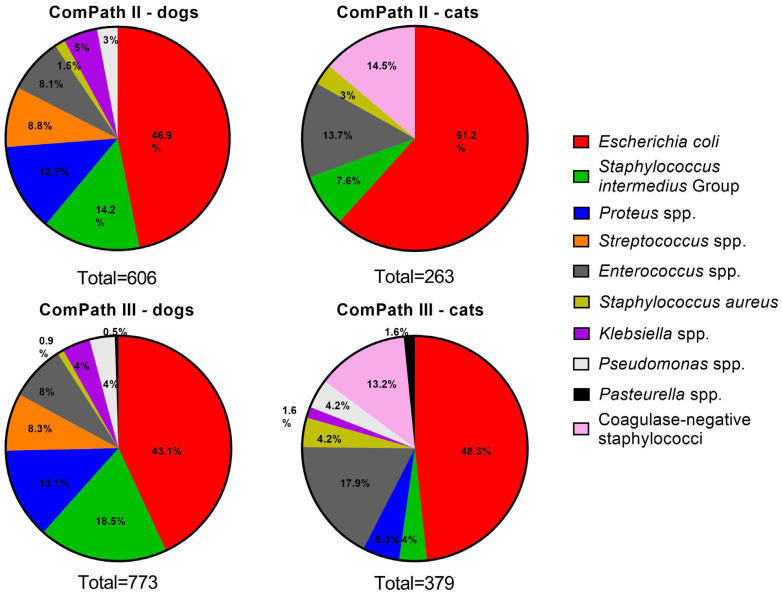
Overview of the isolation percentages of canine and feline UTI bacterial pathogens from diseased animals during the ComPath II (2013–2014) and III (2017–2018) programmes.

Figure 2 gives an overview of the isolated canine and feline UTI pathogens across the two ComPath programmes. *Escherichia coli* was the most frequently isolated bacterium in UTI in both dogs and cats and across the two collection periods: 46.9% (n = 284 in dogs, ComPath II); 61.2% (n = 161 in cats, ComPath II); 43.1% (n = 333 in dogs, ComPath III); and 48.3% (n = 183 in cats, ComPath III). The frequency of the other isolated pathogens also showed cross-programme consistency (see below).

#### 2.1.2. Dogs—ComPath II

Next to *E. coli*, *Staphylococcus intermedius* Group was the second-most frequently isolated species (n = 86, 14.2%), followed by *Proteus mirabilis* (n = 77, 12.7%). Fifty-three (8.8%) *Streptococcus* spp. were present, which included *Streptococcus* (*S*.) *canis* (n = 36), *S. castoreus* (n = 11), *S. dysgalactiae* (n = 5), and *S. equi* (n = 1). Other isolated Gram-positive bacteria included *Staphylococcus aureus* (n = 9, 1.5%, all methicillin-susceptible *S. aureus* (MSSA)) and the more frequent *Enterococcus* spp. (n = 49, 8.1%), of which *E. faecalis* was the most prevalent (n = 32), followed by *E. casseliflavus* (n = 5), *E. faecium* (n = 5), *E. canintestini* (n = 3), *E. cecorum* (n = 1), *E. gallinarum* (n = 1), *E. hirae* (n = 1), and *E. mundtii* (n = 1). Finally, *Klebsiella pneumoniae* and *Pseudomonas aeruginosa* were present in 5.0% (n = 30) and 3.0% (n = 18) of the samples, respectively.

#### 2.1.3. Cats—ComPath II

Coagulase-negative staphylococci were the second-most frequently isolated species from feline urinary samples (n = 38, 14.5%), including *Staphylococcus felis* (n = 17), *S. epidermidis* (n = 7), *S. saprophyticus* (n = 3), *S. sciuri* (n = 2, although in this study still considered a *Staphylococcus* spp., this organisms was recently reclassified as *Mammaliicoccus sciuri*), *S. xylosus* (n = 2), *S. condimenti* (n = 1), *S. haemolyticus* (n = 1), *S. hominis* (n = 1), *S. pasteuri* (n = 1), and *S. warneri* (n = 1), together with *Enterococcus* spp. (n = 36, 13.7%), which included *E. faecalis* (n = 31), *E. faecium* (n = 4), and *E. casseliflavus* (n = 1) [15]. Other isolated Gram-positive bacteria included *S. aureus* (n = 8, 3.0%, 1 methicillin-resistant *S. aureus* (MRSA)) and bacteria from the *S. intermedius* Group (n = 20, 7.6%).

#### 2.1.4. Dogs—ComPath III

In alignment with the ComPath II collection, bacteria from the *S. intermedius* Group were the second-most frequently isolated species from canine urinary samples (n = 143, 18.5%), followed by *Proteus* spp. (n = 101, 13.1%). The latter included *Proteus mirabilis* (n = 100) and *Proteus vulgaris* (n = 1). Other frequent isolates were *Streptococcus* spp. (n = 64, 8.3%) and *Enterococcus* spp. (n = 62, 8.0%). More detailed, the species were *S. canis* (n = 63), *S. dysgalactiae* (n = 1), *E. faecalis* (n = 49), *E. faecium* (n = 6), *E. canis* (n = 3), *E. gallinarum* (n = 2), and *E. hirae* (n = 2). Less prevalent bacteria were *Klebsiella* spp. (n = 31, 4.0%), including *Klebsiella pneumoniae* (n = 26), *Klebsiella variicola* (n = 3), and *Klebsiella oxytoca* (n = 2); *Pseudomonas* spp. (n = 28, 3.6%), including *Pseudomonas aeruginosa* (n = 22), *Pseudomonas monteilii* (n = 1), *Pseudomonas putida* (n = 1), and four non-speciated isolates; *S. aureus* (n = 7, 0.9%, 1 MRSA); and finally *Pasteurella* spp. (n = 4, 0.5%), including *Pasteurella dagmatis* (n = 3) and *Pasteurella multocida* (n = 1).

#### 2.1.5. Cats—ComPath III

In concert with the cross-programme consistency of the isolation results of dog samples, the relative frequencies of the cat UTI pathogens were coherent between ComPath II and III. As mentioned above, *E. coli* was most frequently isolated. *Enterococcus* spp. followed second (n = 67, 17.7%) and coagulase-negative staphylococci third (n = 50, 13.2%). The former included *E. faecalis* (n = 57), *E. faecium* (n = 7), *E. casseliflavus* (n = 2), and *E. hirae* (n = 1); the latter, *S. felis* (n = 32), *S. epidermidis* (n = 7), *S. haemolyticus* (n = 2), *S. sciuri* (n = 2), *S. warneri* (n = 2), *S. caprae* (n = 1), *S. hominis* (n = 1), *S. nepalensis* (n = 1), *S. saprophyticus* (n = 1), and one non-speciated isolate. The occurrence of the other pathogens *Proteus* spp., *Pseudomonas* spp., *S. aureus*, *S. intermedius* Group, *Klebsiella* spp., and *Pasteurella* spp. was 5.3% (n = 20), 4.2% (n = 16), 4.2% (n = 16, 1 MRSA), 4.0% (n = 15), 1.6% (n = 6), and 1.6% (n = 6), respectively. The species genera included *Proteus mirabilis* (n = 18), *Proteus hauseri* (n = 1), *Pseudomonas aeruginosa* (n = 15), *Pseudomonas monteilii* (n = 1), *Klebsiella pneumoniae* (n = 4), *Klebsiella variicola* (n = 1), *Klebsiella oxytoca* (n = 1), *Pasteurella multocida* (n = 4), and *Pasteurella dagmatis* (n = 2) (one *Proteus* isolate was non-speciated).

### 2.2. Antimicrobial Susceptibility

The percentages of S, I, and R (when clinical breakpoints were available) and the MIC_50_ and MIC_90_ values for the UTI pathogens *E. coli* (dog and cat), *Proteus* spp. (dog and cat ComPath III, only dog ComPath II), *Pseudomonas* spp. (dog and cat ComPath III, only dog ComPath II), *Klebsiella* spp. (dog), *S. intermedius* Group (dog and cat), *Streptococcus* spp. (dog), *Enterococcus* spp. (dog and cat), and coagulase-negative staphylococci (cat) are summarized in Table 1, Table 2, Table 3 and Table 4. Table 1 and Table 2 summarize the results of the Gram-negative bacteria from ComPath II and ComPath III, respectively. Table 3 and Table 4 summarize the results of the Gram-positive bacteria from ComPath II and ComPath III, respectively. Please note that isolates of which the frequency was below 10 are not reported in the tables. All clinical breakpoints used were the most up to date according to CLSI and species specific and, if applicable, indication specific. Exceptions are the breakpoints used for oxacillin (dogs and cats), cefalexin (cats), doxycycline (dogs and cats for *Streptococcus* spp. and *Enterococcus* spp. and cats for Enterobacterales), trimethoprim-sulfamethoxazole (dogs and cats), and gentamicin (cats and dogs for *Staphylococcus* spp. and cats only for Enterobacterales), which were derived from human medicine.

Additionally, the distributions of MICs for the two most frequently isolated bacteria in UTIs from dogs and cats for ComPath II and ComPath III are given in Supplementary Tables S1–S8: *E. coli* (Table S1) and *S. intermedius* Group (Table S2) from dogs for ComPath II, *E. coli* (Table S3) and coagulase-negative staphylococci (Table S4) from cats for ComPath II, *E. coli* (Table S5) and *S. intermedius* Group (Table S6) from dogs for ComPath III, and *E. coli* (Table S7) and *Enterococcus* spp. (Table S8) from cats for ComPath III.

#### 2.2.1. Antimicrobial Susceptibility of Gram-Negative Pathogens

##### Canine Isolates

**Figure 3:**
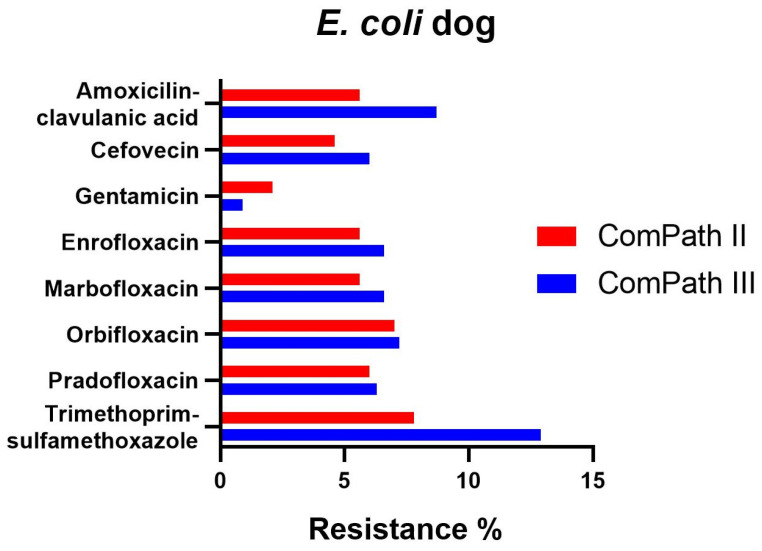
Comparison between resistance levels for canine *E. coli* isolates in ComPath II and ComPath III.

The percentage of resistance in *E. coli* for all antibiotics with clinical breakpoints available was low across both programmes (Table 1 and Table 2), with the exception of trimethoprim-sulfamethoxazole, enrofloxacin, and marbofloxacin in ComPath III, which was moderate (12.9%, 10.5%, 11.4%), and the extremely high resistance against doxycycline (98.8%, only tested in ComPath III). A graphical comparison between resistance levels in ComPath II and ComPath III for *E. coli* is given in Figure 3. For antibiotics without clinical breakpoints (i.e., amoxicillin, cefadroxil, cephalothin, and neomycin), the MIC_50_ and MIC_90_ were identical or similar across programmes.

**Figure 4:**
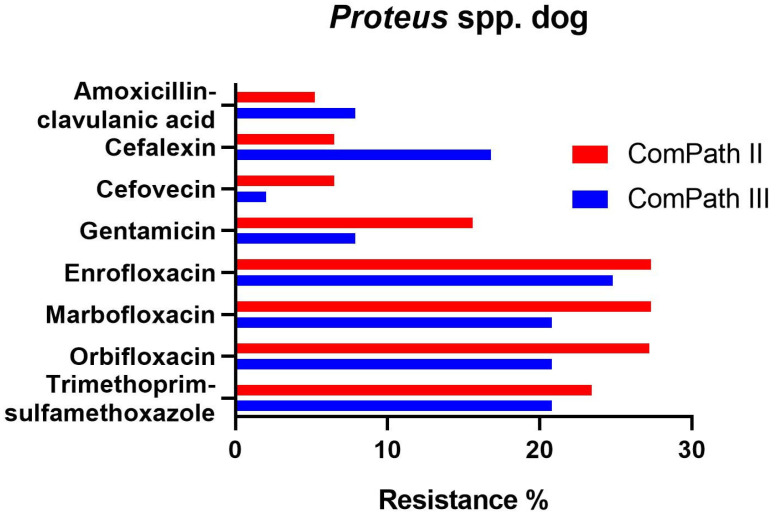
Comparison between resistance levels for canine *Proteus* spp. isolates in ComPath II and ComPath III.

*Proteus* resistance levels ranged from 5.2% against amoxicillin-clavulanic acid to 27.3% against enrofloxacin and marbofloxacin in ComPath II (Table 1). The results were relatively consistent across programmes. However, resistance of *Proteus* against cefovecin (6.5% → 2.0%), gentamicin (15.6% → 7.9%), enrofloxacin (27.3% → 24.8%), marbofloxacin (27.3% → 20.8%), orbifloxacin (27.2% → 20.8%), and trimethoprim-sulfamethoxazole (23.4% → 20.8%) was numerically lower in the ComPath III programme compared to the previous collection period. All strains tested in ComPath III were resistant against doxycycline. Conversely, cefalexin resistance was higher in the ComPath III programme (16.8% vs. 6.5%). The results are summarized in Figure 4. For antibiotics without clinical breakpoints (i.e., amoxicillin, cefadroxil, cephalothin, neomycin, and pradofloxacin), the MIC_50_ and MIC_90_ were identical or similar across programmes.

For *P. aeruginosa*, there was no clinical resistance against gentamicin in both programmes but levels of fluoroquinolone resistance (i.e., enrofloxacin and marbofloxacin) were extremely high (Table 1 and Table 2). *Pseudomonas* showed very high MIC values for amoxicillin (with or without clavulanic acid) and cephalosporines (MIC_50_ and MIC_90_ > 32 or > 64 µg/mL), which is in line with *Pseudomonas*’ intrinsic resistance against beta-lactam antibiotics. MIC_50_ and MIC_90_ values for the other tested antibiotics (neomycin, orbifloxacin, pradofloxacin, and trimethoprim-sulfamethoxazole) were identical or similar across the two programmes.

**Figure 5:**
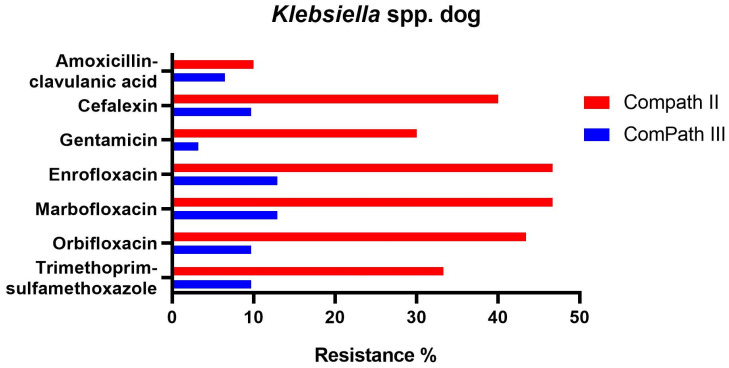
Comparison between resistance levels for canine *Klebsiella* spp. isolates in ComPath II and ComPath III.

For *Klebsiella* spp. pathogens isolated during the ComPath III collection period (Table 2), all but doxycycline (100% resistance) presented low to moderate resistance rates against amoxicillin-clavulanic acid (6.5%), cefalexin (9.7%), gentamicin (3.2%), enrofloxacin (12.9%), marbofloxacin (12.9%), orbifloxacin (9.7%), and trimethoprim-sulfamethoxazole (9.7%). Interestingly, when comparing with the ComPath II programme (Table 1), resistance levels against the other aforementioned antibiotics numerically decreased substantially (cefalexin: −30.3%; gentamicin: −26.8%; enrofloxacin: −33.8%; marbofloxacin: −33.8%; orbifloxacin: −33.6%; trimethoprim-sulfamethoxazole: −23.6%). A comparison between resistance levels in ComPath II and ComPath III for *Klebsiella* is given in Figure 5. For antibiotics without clinical breakpoints (i.e., amoxicillin, cefadroxil, cefovecin, cephalothin, neomycin, and pradofloxacin), the MIC_50_ values were identical or similar across programmes. A decrease in MIC_90_ values from ComPath II to ComPath III was demonstrated for cefovecin (>32 µg/mL → 2 µg/mL), cephalothin (>64 µg/mL → 16 µg/mL), neomycin (16 µg/mL → 1 µg/mL), and pradofloxacin (4 µg/mL → 0.25 µg/mL).

##### Feline Isolates

**Figure 6:**
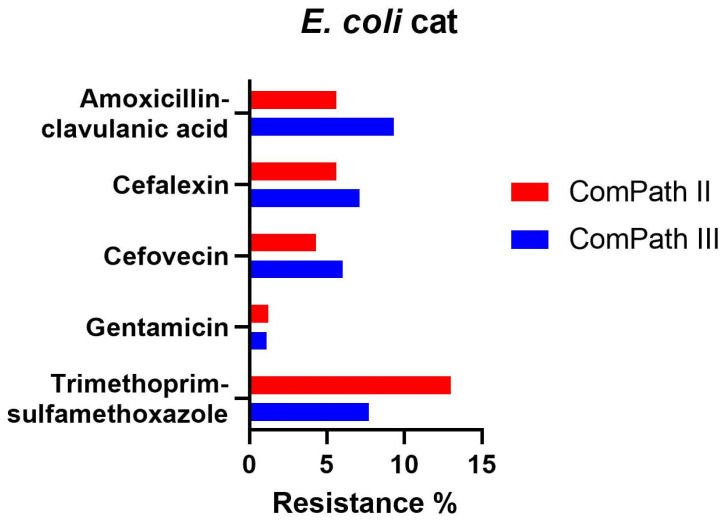
Comparison between resistance levels for feline *E. coli* isolates in ComPath II and ComPath III.

Resistance levels in *E. coli* isolated during ComPath III from cats were low (Table 2): amoxicillin-clavulanic acid 9.3%; cefalexin 7.1%; cefovecin 6.0%; gentamicin 1.1%, and trimethoprim-sulfamethoxazole 7.7%. These results were comparable with the MIC results from the previous programme (Table 1). A comparison between resistance levels in ComPath II and ComPath III for *E. coli* is given in Figure 6. For antibiotics without clinical breakpoints (i.e., amoxicillin, cefadroxil, cephalothin, neomycin, enrofloxacin, marbofloxacin, orbifloxacin, and pradofloxacin), the MIC_50_ and MIC_90_ were identical or similar across programmes, with the exception of a decrease in MIC_90_ values for the fluoroquinolones from ComPath II to ComPath III: enrofloxacin (0.5 µg/mL → 0.06 µg/mL), marbofloxacin (0.5 µg/mL → 0.06 µg/mL), orbifloxacin (2 µg/mL → 0.25 µg/mL), and pradofloxacin (0.25 µg/mL → 0.03 µg/mL).

*Proteus* was isolated from cats only in the ComPath III programme (Table 2). Resistance against amoxicillin-clavulanic acid, gentamicin, and trimethoprim-sulfamethoxazole was 15.0%, 10.0%, and 25.0%, respectively, and relatively comparable with the results obtained in dogs (5.9%, 7.9%, and 20.8%). For cefalexin and cefovecin, the resistance rates for cats were higher compared to dogs (35.0% vs. 16.8% and 15.0% vs. 2.0%). Analogous to dogs, doxycycline resistance was extremely high (90%). MIC_50_ values for amoxicillin, cefadroxil, cephalothin, neomycin, and pradofloxacin were identical or similar for cats and dogs in the ComPath III programme. The MIC_90_ value was higher for cats than for dogs in ComPath III for cephalothin (dogs: 16 µg/mL, cats: >64 µg/mL), and higher for dogs than for cats for neomycin (dogs: 32 µg/mL, cats: 8 µg/mL) and for pradofloxacin (dogs: 4 µg/mL, cats: 1 µg/mL).

Like *Proteus*, *Pseudomonas* was only isolated from cats in the ComPath III programme (Table 2). Resistance against gentamicin for *P. aeruginosa* was non-existent (using the 6th edition VET01S human clinical breakpoints, as the 7th edition no longer has breakpoints for this indication). Dovetailing the results from dogs, the isolates showed very high MIC values for amoxicillin (with or without clavulanic acid) and cephalosporines (MIC_50_ and MIC_90_ >32 or > 64 µg/mL). For the other tested antibiotics (neomycin, enrofloxacin, marbofloxacin, orbifloxacin, pradofloxacin, and trimethoprim-sulfamethoxazole), MIC_50_ values were identical and MIC_90_ values were similar to the dog values reported in ComPath III.

#### 2.2.2. Antimicrobial Susceptibility of Gram-Positive Pathogens

##### Canine Isolates

In general, resistance levels in most Gram-positive pathogens in dogs in both collection programmes were non-existent and low (1–10%) to moderate (10–20%), with some exceptions. Susceptibility and resistance levels were noticeably stable between both collection periods.

**Figure 7:**
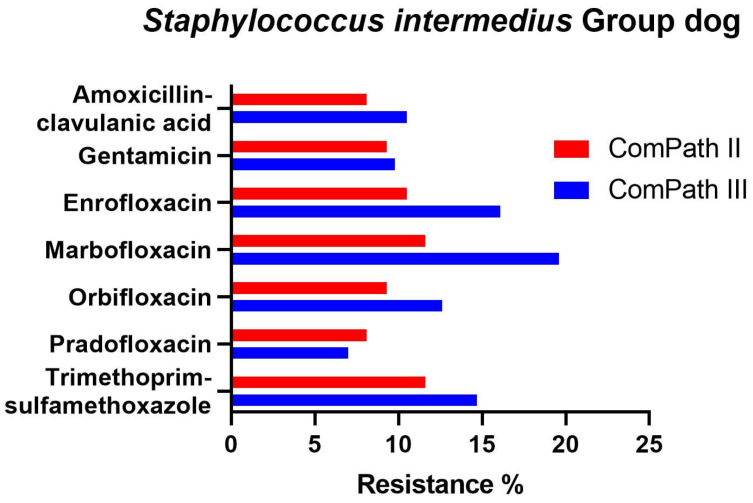
Comparison between resistance levels for canine bacteria from the *S. intermedius* Group in ComPath II and ComPath III.

In bacteria from the *S. intermedius* Group collected during the ComPath III programme (Table 4), resistance levels were low against amoxicillin-clavulanic acid (9.1%), gentamicin (9.8%), and pradofloxacin (7.0%), and moderate against enrofloxacin (16.1%), marbofloxacin (19.6%), orbifloxacin (12.6%), and trimethoprim-sulfamethoxazole (14.7%). Results were similar for bacteria from the *S. intermedius* Group isolated during ComPath II (Table 3), except for orbifloxacin where the resistance level could be classified as low (9.3%), although the difference in resistance prevalence with the more recent programme was only 3.3%. A comparison between resistance levels in ComPath II and ComPath III for the *S. intermedius* Group is given in Figure 7. A high proportion of intermediate strains were detected for enrofloxacin and marbofloxacin in both programmes (45.3% and 67.5% in ComPath II and 43.4% and 62.2% in ComPath III). For antibiotics (tested in both programmes) without clinical breakpoints (i.e., amoxicillin, cefadroxil, cefalexin, cefovecin, cephalothin, and neomycin), the MIC_50_ and MIC_90_ values were identical or similar across programmes, except for a clear increase in MIC_90_ value from ComPath II to ComPath III for amoxicillin (0.25 µg/mL → 16 µg/mL).

*Streptococcus* spp. showed zero to very low resistance rates against enrofloxacin (0%; 0%), marbofloxacin (1.6%; 1.9%), and orbifloxacin (1.6%; 0%) in ComPath III and ComPath II, respectively. Of note were the high proportion of intermediate strains, with percentages ranging from 20.3% against marbofloxacin to 100% against orbifloxacin. Intriguingly, for marbofloxacin, there was a shift from mostly intermediate strains in ComPath II (62.3%) to mostly susceptible strains (78.1% S, 20.3% I) in ComPath III, with resistance levels remaining constant. Resistance to doxycycline amounted to 35.9% in ComPath III. For antibiotics (tested in both programmes) without clinical breakpoints (i.e., amoxicillin, cefadroxil, cefalexin, cefovecin, cephalothin, gentamicin, neomycin, pradofloxacin, and trimethoprim-sulfamethoxazole), the MIC_50_ and MIC_90_ were identical or similar across programmes, except for a decrease from ComPath II to ComPath III for gentamicin (MIC_50_: 32 µg/mL → 4 µg/mL, MIC_90_: 32 µg/mL → 8 µg/mL) and for neomycin (MIC_50_: 128 µg/mL → 32 µg/mL, MIC_90_: >128 µg/mL → 64 µg/mL).

Resistance for *Enterococcus* spp. against amoxicillin-clavulanic acid was low (9.7%, ComPath III) to moderate (14.3%, ComPath II) and moderate against doxycycline (12.9%). *Enterococcus* spp. isolates showed high MIC values for cephalosporines (MIC_50_ ≥ 32 µg/mL and MIC_90_ > 32 µg/mL) and for aminoglycosides (MIC_50_ ≥ 16 µg/mL and MIC_90_ > 32 µg/mL). MIC_50_ and MIC_90_ were identical or similar across programmes, except for a clear decrease in MIC_90_ value from ComPath II to ComPath III for amoxicillin (>64 µg/mL → 4 µg/mL).

##### Feline Isolates

Overall, resistance levels for *S. intermedius* Group were higher for bacteria isolated from cats when compared to dogs. Resistance in the ComPath III collection (Table 4) ranged from 13.3% against gentamicin to 26.7% for amoxicillin-clavulanic acid. Compared with the ComPath II programme (Table 3), resistance against amoxicillin-clavulanic acid and gentamicin increased by 16.7% and 3.3%, respectively. For the other antibiotics, the MIC_50_ and MIC_90_ values were identical or similar across programmes, except for an increase in MIC_50_ and MIC_90_ value from ComPath II to ComPath III for amoxicillin (MIC_50_: 0.12 µg/mL → 0.5 µg/mL, MIC_90_: 2 µg/mL → 64 µg/mL), an increase in MIC_90_ value for cefadroxil (4 µg/mL → >32 µg/mL), and a decrease in MIC_50_ value for neomycin (8 µg/mL → 0.25 µg/mL).

Resistance for *Enterococcus* spp. against amoxicillin-clavulanic acid was absent (ComPath II) to low (7.5%, ComPath III), whereas the resistance to doxycycline amounted to 11.9%. Analogous to dog isolates, *Enterococcus* spp. isolated from urine samples from cats displayed very high MIC_50_ and MIC_90_ values for cephalosporines and aminoglycosides (Table 3 and Table 4). MIC_50_ and MIC_90_ were identical or similar across the ComPath II and ComPath III programmes, except for a clear increase in MIC_90_ value from ComPath II to ComPath III for trimethoprim-sulfamethoxazole (2 µg/mL → >16 µg/mL).

For coagulase-negative staphylococci, resistance levels against amoxicillin-clavulanic acid, gentamicin, and trimethoprim-sulfamethoxazole in ComPath III (Table 4) were 10.0%, 6.0%, and 2.0%, respectively. Results for gentamicin were comparable with results obtained in ComPath II (2.6%; Table 3). Resistance against amoxicillin-clavulanic acid and trimethoprim-sulfamethoxazole was not present in isolates from ComPath II. For the other antibiotics, the MIC_50_ and MIC_90_ values were identical or similar across programmes, except for an increase in MIC_90_ value from ComPath II to ComPath III for amoxicillin (0.5 µg/mL → 4 µg/mL).

### 2.3. Methicillin-Resistant Staphylococci

Of the 40 isolated *S. aureus* bacteria in total (i.e., nine canine and eight feline isolates from ComPath II and seven canine and sixteen feline isolates from ComPath III), three (7.5%) were MRSA (i.e., one feline isolate from ComPath II, and one canine and one feline isolate from ComPath III), which were all *mecA* positive. Of the total number of bacteria belonging to the *S. intermedius* Group (n = 264) and coagulase-negative staphylococci (n = 88), 11.4% and 18.2% were methicillin resistant, respectively. Of these, 56.1% and 37.5% were *mecA* positive.

### 2.4. Multidrug Resistance

**Figure 8:**
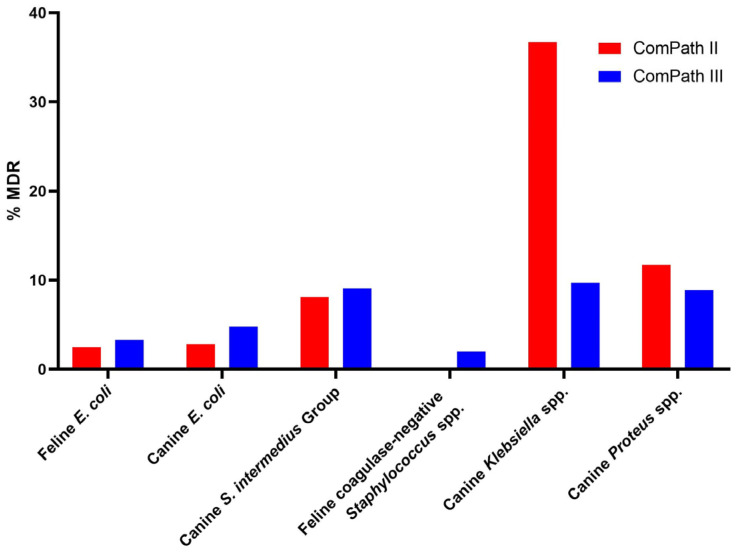
Summary and comparison of MDR across ComPath II and ComPath III for *E. coli* isolates from cats and dogs, canine *S. intermedius* Group isolates, feline coagulase-negative *Staphylococcus* spp., and canine *Klebsiella* and *Proteus* spp.

The multidrug resistance (MDR) results and comparison between the two programmes are summarized in Figure 8. Sufficient clinical breakpoints (>3 for antimicrobials from different classes) to evaluate MDR were only available for *E. coli* isolates from cats and dogs, canine *S. intermedius* Group isolates, feline coagulase-negative *Staphylococcus* spp., and canine *Klebsiella* and *Proteus* spp.

## 3. Discussion

The objective of this study was to determine the levels of antimicrobial susceptibility of UTI pathogens isolated from companion animals in 2013–2014 and 2017–2018 against a range of commonly used veterinary antimicrobial agents and to discern possible temporal trends.

In alignment with other European studies regarding companion animal UTIs, *E. coli* was the most frequently isolated bacterium in dogs and cats with UTI [16,17,18]. The *E. coli* predominance in companion animal UTI is also observed in other parts of the world, e.g., China, the United States of America, and Australia [19,20,21,22]. Comparably, *E. coli* is the most important pathogen for urinary infections in humans [23]. In contrast to studies from Italy and Portugal, the second most frequently isolated bacteria from canine urinary samples belonged to the *S. intermedius* Group, closely followed by *Proteus* spp. (in the studies from Italy and Portugal, *Proteus* spp. were the second most frequently isolated) [17,18]. In cats, the second most frequently isolated pathogens were *Enterococcus* spp. and (or closely followed by in ComPath III) coagulase-negative staphylococci. This is again in alignment with recent investigations [16,18,24,25].

The identified *Staphylococcus* species varied significantly between dogs and cats. Bacteria from the *S. intermedius* Group were more prevalent in dogs, whereas coagulase-negative staphylococci were more dominant in feline UTI infections. In agreement with other studies, *Staphylococcus felis*, belonging to the coagulase-negative staphylococci, was frequently detected in urine samples of cats with UTI [16,26].

Principally, the resistance levels for canine and feline UTI pathogens against most tested antibiotics with clinical breakpoints available were low and relatively comparable across the two ComPath programmes, with some exceptions. The results also generally dovetail what has been reported in the ComPath I programme, which ran from 2008 until 2010 [12].

*E. coli* resistance was low (<10%) for most tested antibiotics across both programmes, except for moderate levels of resistance against trimethoprim-sulfamethoxazole in cat isolates in ComPath II (13.0%) and dog isolates in ComPath III (12.9%) and enrofloxacin (10.5%) and marbofloxacin (11.4%) resistance in canine isolates of ComPath III. This aligns well with the high susceptibility percentages of *E. coli* against fluoroquinolones and trimethoprim-sulfamethoxazole reported in the ComPath I programme, indicating a stable temporal resistance pattern in veterinary UTI E. coli pathogens [12]. Our *E. coli* results are largely comparable with the results obtained from the German monitoring programme over the 2008–2021 period, although 100% resistance against ampicillin is reported in feline isolates (but not in canine) [27]. In contrast, the SWEDRES-SVARM programme of Sweden only reports around 15–20% of ampicillin resistance in feline *E. coli* UTI isolates [28]. The French national surveillance programme ResaPath reports a gradual increase in amoxicillin and amoxicillin-clavulanic acid resistance in canine UTI *E. coli* ranging from approximately 20 to 30%, which is not reflected in our results [29]. However, an unbiased comparison is difficult as the ResaPath programme applies different antimicrobial susceptibility testing (AST) techniques and uses breakpoints established by the Comité de l’Antibiogramme de la Société Française de Microbiologie which may be different from the CLSI breakpoints.

When applying the CLSI amoxicillin-clavulanic acid clinical breakpoints that were available at the time of the ComPath II (0.25/1 µg/mL) and ComPath III (8 µg/mL) programmes, this led to an extremely high level of resistance (99.4%) against amoxicillin-clavulanic acid in *E. coli* isolated from UTI from cats in ComPath II compared to the low resistance level (9.3%) in the ComPath III programme, despite the similar MIC_50_ (4 µg/mL) and MIC_90_ (8 µg/mL) values. As discussed by other authors, the high level of resistance in the ComPath II programme is an anomaly created by inadequate feline clinical breakpoint setting [12,30]. The Veterinary Antimicrobial Susceptibility Testing (VAST) Working Group of CLSI, due to a lack of specific feline urinary PK/PD data, set the UTI breakpoints conservatively based on skin and soft tissue infection PK/PD (S ≤ 0.25 mg/mL and R ≥ 1 µg/mL). However, with the availability of new urinary PK data, the clinical breakpoint has been revised to S ≤ 8 µg/mL, which corresponds to the canine and human clinical breakpoint [31]. This was the breakpoint used in this paper and led to a marked reduction in previously overestimated resistance. This emphasizes (1) the need to note the exact clinical breakpoint that was used during the review of the data and (2) the need for feline-specific and infection-specific breakpoints [30].

*Proteus* spp. resistance against trimethoprim-sulfamethoxazole (approximately 20%) was similar in both programmes for dogs (*Proteus* spp. was not isolated in ComPath II for cats) and aligns well, together with ComPath III feline results with recent studies with isolates from the US and central Italy [18,21]. Fluoroquinolone resistance in canine isolates was high and for enrofloxacin there was a high proportion of intermediate strains (66.3–71.4%). In the ComPath I programme, susceptibility percentages against fluoroquinolones and trimethoprim-sulfamethoxazole were higher (ranging from 81.9–91.7%), but as previously mentioned for the fluoroquinolones, the difference in numbers is difficult to assess due to the use of different breakpoints [12].

*P. aeruginosa* is notoriously resistant (intrinsically and acquired) to a large number of currently available antibiotics [32,33]. In this study, this is exemplified by the high value (32–>64) of the MIC_50_ and MIC_90_ for most of the tested antibiotics such as amoxicillin-clavulanic acid, first generation cephalosporins, tetracyclines, or cefovecin, with the exceptions being some fluoroquinolones and aminoglycosides. Also, *P. aeruginosa* is typically intrinsically resistant to trimethoprim and sulfonamides [34]. The lower MIC_50_ and MIC_90_ values for fluoroquinolones and aminoglycosides for *P. aeruginosa* were also observed in a US study on canine urine samples submitted to a veterinary diagnostic laboratory in Illinois [21]. Additionally, susceptibility rates against gentamicin of approximately 90% were observed in canine *P. aeruginosa* isolates. These high levels of sensitivity against gentamicin are in agreement with other studies in dogs in the US and cats in Australia and the Norwegian national monitoring program NORM-VET [21,26,35]. This indicates geographical consistency in *Pseudomonas* resistance and susceptibility. In contrast to the high gentamicin susceptibility, there was extremely high resistance against marbofloxacin and enrofloxacin in both programmes.

Fluoroquinolone resistance against enrofloxacin, marbofloxacin, orbifloxacin, and the member of the newer generation pradofloxacin was low to moderate across both programmes for most Gram-negative and Gram-positive pathogens, with the exception of high levels of resistance in *Proteus* (for dogs), extremely high resistance levels in *Pseudomonas* against enrofloxacin (ComPath II and III) and marbofloxacin (ComPath II and III), and high resistance in *Klebsiella* isolates from dogs during the ComPath II programme. In contrast, *Klebsiella* isolated from UTI in dogs during the ComPath III programme were mostly susceptible (resistance 9.7–12.9%).

ComPath II isolates of the Enterobacteriaceae family that were resistant to ampicillin or amoxicillin and that via subsequent testing belonged to the non-wild type part of the MIC distribution for cefotaxime and ceftazidime were also screened for extended-spectrum beta-lactamases (ESBLs) and plasmid-mediated cephalosporinases (pAmpC), which has been described in-depth in a separate paper [36]. Of the 63 sequenced isolates, 53 isolates harboured a *bla*_ESBL_ or *bla*_AmpC_ gene.

In alignment with the MIC and resistance results obtained for *E. coli*, resistance levels of bacteria from the *S. intermedius* Group against the tested antibiotics with clinical breakpoints available was low to moderate and consistent across programmes, with the exception of high (26.7%) resistance levels for feline isolates against amoxicillin-clavulanic acid. Overall, resistance levels from feline isolates were slightly higher than those from canine isolates, which could be attributable to the lower sample size of the isolates compared to dogs. The canine isolate resistance prevalence corresponds well with the results obtained in the ComPath I programme (collection period 2008–2010), where resistance levels for trimethoprim-sulfamethoxazole and several fluoroquinolones ranged from 3–6%, again indicating a fairly stable temporal resistance pattern [12].

During the time of writing of this manuscript, a new edition (the 7th) of the VET01S document of the CLSI was published, with several updates to clinical breakpoints for different species and indications [37]. One of the major revisions was the change in breakpoints for enrofloxacin and marbofloxacin in dogs, which changed from 0.5/4 and 1/4 to 0.06/0.5 and 0.12/0.5, for *Staphylococcus* spp., *E. coli*, *Klebsiella* spp., *Proteus* spp., and *P. aeruginosa*. The breakpoints were revised via pharmacokinetic modelling and considering protein binding, Monte Carlo simulation (MCS), and probability of target attainment analysis (PTA) [38]. Although the change had a minor impact in the resistance levels of Gram-negative bacteria (except for an average 10% increase in resistance for *Proteus*), the classification of isolates from the *Staphylococcus intermedius* Group as susceptible, intermediate, or resistant was majorly reshuffled (Table 5). Additionally, the I category was replaced by the new susceptible-dose dependent (SDD) category, indicating that the susceptibility of an isolate depends on the dosage regimen that is used in the patient [37]. While resistance levels increased slightly, the overwhelming proportion of susceptible strains is now cut in half with approximately equal halves of S and I for enrofloxacin and a larger proportion of I compared to S for marbofloxacin. For surveillance and monitoring purposes, this could lead to a biased interpretation of the increase in intermediate resistance if one does not take into account the change in breakpoints.

Caution should be exercised when interpreting these new breakpoints. First, although breakpoints, and thus classification in S, I, and R, have changed, the MIC distribution and MIC_50_ and MIC_90_ values have remained relatively stable over time, indicating no major fluctuation in susceptibility or resistance. Second, the setting of a clinical breakpoint in the ideal scenario should take into account the MIC distribution of the pathogen, pharmacokinetic-pharmacodynamic (PK-PD) considerations, and clinical outcome data [39,40]. As is often the case in veterinary medicine, clinical data were not available for these bug–drug combinations [41]. Finally, a 24 h fAUC:MIC ratio of 72 was used as PK-PD target based on previous recommendations. However, this value is tentative as it has been shown that the magnitude of the PK-PD target can be drug, pathogen, and species specific [42].

Interestingly, relatively high percentages of *Streptococcus* isolates were classified as intermediate or susceptible for fluoroquinolones. In ComPath II, the prevalence of intermediate strains was 56.6%, 62.3%, and 100.0% for enrofloxacin, marbofloxacin, and orbifloxacin, respectively. In ComPath III, these results were fairly similar, except for the lower number of intermediate strains for marbofloxacin (and a subsequent higher number of susceptible strains, as also seen in ComPath I): 51.6% (enrofloxacin), 20.3% (marbofloxacin), and 95.3% (orbifloxacin). Increased intermediate percentages for some fluoroquinolones and *Streptococcus canis* were also reported in the ComPath I programme (28.6% for enrofloxacin and 85.7% for orbifloxacin), whereas marbofloxacin showed a susceptibility of 91.4% [12]. The variability of the proportion between susceptible and intermediate strains across programmes can be explained by the clustering of the *Streptococcus* population around the clinical breakpoint of susceptibility. The intermediate category implies that therapeutic efficacy can be achieved in body sites where the drug accumulates or when higher doses can be used. In alignment with this definition, European Committee on Antimicrobial Susceptibility Testing (EUCAST) changed the name and definition of “I” from “intermediate” to “susceptible, increased exposure” [43].

Similar to *Pseudomonas* spp., *Enterococcus* spp. are intrinsically resistant to a large number of antibiotics, e.g., β-lactams, aminoglycosides, lincosamides, streptogramins, and trimethoprim-sulfamethoxazole, corroborated by the very high MIC_50_ and MIC_90_ values for β-lactams (cephalosporine values where higher than amoxicillin values) and aminoglycosides [44]. Although *Enterococcus* is intrinsically resistant to trimethoprim-sulfamethoxazole, low MIC_50_ values were observed for both canine (0.03 µg/mL) and feline (0.06 µg/mL) isolates in the two programmes. However, this is an experimental artifact, as *Enterococcus* spp. can absorb folic acid from the environment, thereby bypassing the effects of potentiated sulphonamides [44]. When performing antimicrobial susceptibility testing in a medium devoid of folate, this will yield susceptible results, which does not translate to treatment of in vivo infections [45,46]. Drugs of choice for the treatment of UTIs of enterococcal aetiology include ampicillin and amoxicillin (with clavulanic acid) [47,48].

Marques et al. investigated possible temporal trends in resistance against commonly used antibiotics in canine and feline UTI pathogens in Portugal (a country where no data were collected from during the two ComPath programmes) from 1999–2014 [16]. The study showed strong increases in resistance against most antibiotics (amoxicillin-clavulanic acid, trimethoprim-sulfamethoxazole, fluoroquinolones, gentamicin, and tetracycline) in Enterobacterales, exemplified by a three- and four-fold increase in resistance against amoxicillin-clavulanate and third generation cephalosporins, respectively, in *E. coli*, resulting in resistance levels around 30–40%. Resistance levels for these molecules in *E. coli* obtained in our two studies were generally lower, and no major discernible increase in resistance against most tested antibiotics of the 2013–2018 time period was observed. Caution should be exercised when interpreting temporal trends from these data over a limited period. More observations are needed.

There is a substantial paucity in clinical breakpoints for veterinary pathogens and indications [12]. Looking at our data, of the 42 bug–drug combinations for Gram-positive pathogens and 70 combinations for Gram-negative bacteria from dogs, only 31.0% (13/42) and 42.9% (30/70) were covered by clinical breakpoints, respectively, some of which even included human breakpoints. For cats, these percentages were even lower, 16.7% (7/42) and 24.3% (17/70). At the moment, there is only one standards-setting organization that develops veterinary-specific criteria, the CLSI subcommittee on Veterinary Antimicrobial Susceptibility Testing (CLSI-VAST), and a limited number of species- and indication-specific interpretive criteria in veterinary medicine have been published [49]. More recently (2015), a EUCAST veterinary subcommittee has been established and is working on veterinary-specific clinical breakpoints [50,51]. It remains to be seen what the impact will be of two organizations responsible for breakpoint setting on the harmonization of the published clinical breakpoints.

There are however some limitations to this study and by extension to the CEESA surveillance programmes in general, as candidly described by de Jong et al. [11]. First, notwithstanding the large number isolates (n = 2021) that were amassed during both collection periods and tested, this still constitutes a very small sample of the total UTI infection incidence in the EU. Second, although temporal patterns in susceptibility and resistance can be discerned across ComPath surveillance programmes, interpretation should be done with caution, as certain bacterial species only had a limited number of isolates. Third, it cannot be excluded that some isolated bacteria were part of the urinary microbiome, and therefore not associated with clinical disease. Fourth, the lack of clinical breakpoints for many bug–drug combinations makes interpretation of the MIC results difficult and does not allow for a clear recommendation to be made to the veterinarian. Finally, the correlation between in vitro susceptibility results and clinical outcomes might not be clear-cut, as some antibiotics can achieve higher urinary concentrations compared to plasma (which is the most common matrix used to determine the PK/PD relationship), and thus still be effective [21].

Nonetheless, despite the abovementioned drawbacks (often inherent to surveillance studies), the ComPath programmes conducted on behalf of the CEESA consortium are for the moment the only surveillance programmes monitoring companion animal (UTI) pathogen resistance on a European scale with high numbers of isolates. However, recently, the European Antimicrobial Resistance Surveillance network in Veterinary medicine (EARS-Vet) was established and the scope of its programme has been defined [14,52]. The goal of this initiative is to monitor resistance levels of eleven bacterial species in six animal species (cattle, swine, chickens, turkeys, cats, and dogs). The scope of this project broadly corresponds with the CEESA surveillance programmes VetPath and ComPath, with some dissimilarities [10]. For example, more bacterial pathogenic species are isolated in the CEESA programmes (including anaerobes). Additionally, another CEESA initiative, the MycoPath programme, is focussed on *Mycoplasma* species, which are not covered in the EARS-Vet scope. Importantly, there are two additional major differences: (1) The AST data in the EARS-Vet programme will be obtained nationally, and therefore MIC determination methodology has the probability of being unharmonized; and (2) AST results will be interpreted according to the ECOFFs [52]. The ECOFF describes the MIC value above which bacterial isolates have phenotypically detectable acquired resistance mechanisms, i.e., microbiological resistance. ECOFFs are potent tools in resistance surveillance programs, however, they often do not give an indication on the therapeutic effectiveness of the investigated drug for the specific pathogen, as is described by the clinical breakpoint [53,54]. ECOFFs are used to set clinical breakpoints, together with PK/PD and clinical outcome data [39]. Therefore, using ECOFFs as a basis, the reporting of non-wild type strains as resistant is misleading.

## 4. Materials and Methods

### 4.1. Bacterial Collection

Samples were collected during 2013–2014 (ComPath II) and 2017–2018 (ComPath III) across 12 European countries: Belgium, Czech Republic, France, Germany, Hungary, Italy, The Netherlands, Poland, Spain, Sweden, Switzerland, and the United Kingdom (UK).

Bacterial isolates were exclusively collected from pet dogs and cats with confirmed clinical UTI and prostate infections. Urine samples for bacterial culture were collected by veterinary professionals via cystocentesis (preferred method), catheterisation, or midstream voided morning urine.

Conforming to the previous ComPath I study, samples were excluded if the host dogs and cats had been treated with antibiotics during the last four weeks prior to sampling and/or if the animals were chronically ill [11,12]. One isolate per bacterial species per sample per pet was exclusively utilised in order to prevent a collection of strains that were epidemiologically related. Additionally, veterinary practitioners completed and supplied a sampling form for each sample collected to confirm compliance with the study protocol.

After sample collection at the veterinary practice or clinic, subsequent isolation, identification, and storage were performed at the local national laboratories. For isolation and identification of the strains, morphological characteristics on selective agar plates and biochemical tests were used. Next, the isolates were shipped to the central laboratory (IHMA Europe Sàrl, Monthey, Switzerland) for storage and MIC testing. Upon arrival at the central laboratory, each isolate was sub-cultured from its transport medium onto an appropriate agar medium. After checking for colonial viability and purity, the resulting overnight growth was used to reidentify each bacterial isolate using matrix-assisted laser desorption/ionization–time of flight (MALDI-TOF) mass spectrometry, after which 1 mL of Tryptic Soy Broth with glycerol 15% (Thermo Fisher Scientific–Oxoid, Waltham, MA, USA) was densely inoculated to prepare freezer stocks. These isolates were subsequently stored at approximately −75 °C.

### 4.2. Antimicrobial Susceptibility Testing

Susceptibility testing of the isolated strains was carried out at the central laboratory using standardised agar dilution methodology (ComPath II) and standardised broth dilution methodology (ComPath III) as recommended by the Clinical and Laboratory Standards Institute [55].

*Enterococcus faecalis* ATCC 29212, *E. coli* ATCC 25922, *E. coli* ATCC 35218, *Pseudomonas aeruginosa* ATCC 27853, *Staphylococcus aureus* ATCC 29213, and *Streptococcus pneumoniae* ATCC 49619 were used as quality control isolates.

The following 15 antibiotics/combinations were tested by means of two-fold serial dilutions: amoxicillin, amoxicillin-clavulanic acid (2:1), cefadroxil, cefovecin, cephalexin, cephalothin, doxycycline (only in the ComPath III programmes), enrofloxacin, gentamicin, marbofloxacin, neomycin, orbifloxacin, oxacillin (only for staphylococci), pradofloxacin, and trimethoprim-sulfamethoxazole (1:19).

For the agar dilution method, a direct suspension of each organism was prepared by selecting isolated colonies from a nonselective agar plate. Turbidity comparable to the McFarland 0.5 standard (1–2 × 10^8^ CFU/mL) was prepared. This suspension was then diluted 1:10 in sterile broth to obtain the desired inoculum of 10^7^ CFU/mL. Next, 0.2 mL of each mixed suspension was transferred to individual wells in a 96 well plate and 1 μL of each inoculum was spotted onto the antimicrobial containing agar plates using a multiple sample inoculator (MAST Uri Dot). The final inoculum delivered to the plate was approximately 10^4^ CFU per spot (i.e., ca. 5 × 10^4^ CFU/mL). This procedure was carried out within 30 min of inoculum preparation.

An antimicrobial free agar plate was inoculated first; then, starting with the lowest concentration of the first antimicrobial, all antimicrobial plates were inoculated. Antimicrobial free plates were inoculated between each antimicrobial compound and at the completion of inoculating to ensure that there was no contamination during inoculation. The plates were allowed to stand at room temperature until the moisture in the inoculum spots had been absorbed into the agar (not longer than 30 min). The MIC agar plates were then incubated at 35 ± 2 °C for 16–24 h in an aerobic atmosphere, except for *Streptococcus* spp. agar plates which were incubated at 35 ± 2 °C for 20–24 h in a CO_2_ atmosphere.

For the microbroth dilution method, bacterial inocula were prepared at ca. 1 × 10^6^ CFU/mL by diluting 0.5 McFarland suspension 100-fold in cation-adjusted Mueller–Hinton broth (CA-MHB) with N-[Tris(hydroxymethyl)-methyl]-2-aminoethane sulfonic acid (TES) for all isolates except *Streptococcus* spp. A 30-fold dilution in CA-MHB supplemented with 5% lysed horse blood was used when testing *Streptococcus* spp. Antibacterial panels containing 50 μL of antibacterial solutions at 2 × the final concentrations were diluted 2-fold with 50 μL of inoculum to give a final inoculum of ca. 5 × 10^5^ CFU/mL and the desired test concentrations of antibacterial agents. The MIC was defined as the lowest concentration of antimicrobial (μg/mL) that completely inhibited growth, disregarding a single colony or a faint haze caused by the inoculum. The MIC values were recorded using Optiscan reporting forms.

### 4.3. MecA Detection in Staphylococcus spp.

Staphylococci found to be oxacillin resistant (MIC ≥ 4 for *S. aureus*, ≥0.5 for *S. intermedius* Group and coagulase-negative staphylococci) were screened for the presence or absence of the *mecA* gene which encodes production of PBP2a according to a PCR method adapted from Zhang and colleagues [37,56].

### 4.4. Data Analyses

MIC data were interpreted according to CLSI breakpoints (VET01S, 7th edition) [37]. The MIC ranges, MIC distributions, MIC_50_, and MIC_90_ values, and, if applicable, percentages of susceptible, intermediate, or resistant were determined for each antimicrobial, each target pathogen species, and each country. The clinical breakpoints used in this study are listed in Table 6. The clinical breakpoints are also indicated by vertical bars in the MIC distribution tables (Supplementary Tables S1–S8). The rate of resistance for each antimicrobial was described as follows: very low (0.1–1%), low (1–10%), moderate (10–20%), high (20–50%), very high (50–70%), and extremely high (>70%), corresponding to the criteria applied by EFSA/ECDC [57]. The MIC_50_ and MIC_90_ values correspond to the lowest concentration of an antimicrobial agent at which growth is inhibited for 50% and 90% of tested strains, respectively [58]. For antibiotics with clinical breakpoints available, the percentages of resistance were compared across programmes. For antibiotics for which no clinical breakpoints are available, MIC_50_ and MIC_90_ were compared across both programmes.

Multidrug resistance (MDR) of an isolate was defined as clinical resistance to at least one agent in three or more antimicrobial classes [49,59]. Tetracyclines were not included in the MDR analysis, as data for doxycycline were only available for ComPath III and not for ComPath II.

## 5. Conclusions

Overall, antimicrobial resistance for most canine and feline UTI pathogens isolated during the ComPath II (2013–2014) and ComPath III (2017–2018) programmes was low (1–10%) to moderate (10–20%). For several pathogens, the lack of CLSI recommended breakpoints for veterinary use remains a bottleneck and hampers interpretation of resistance prevalence and trends.

## Figures and Tables

**Table 1:** Summary table with percentages of susceptible (S), intermediate (I), and resistant (R) Gram-negative UTI isolates from ComPath II from dogs and cats, together with the MIC_50_ and MIC_90_ values (µg/mL).

	*Escherichia coli* (Dog, n = 284/Cat, n = 161)					*Proteus* spp. (Dog, n = 77)					*Pseudomonas* spp. (Dog, n = 18)					*Klebsiella* spp. (Dog, n = 30)				
Antimicrobial Agent	S (%)	I (%)	R (%)	MIC_50_	MIC_90_	S (%)	I (%)	R (%)	MIC_50_	MIC_90_	S (%)	I (%)	R (%)	MIC_50_	MIC_90_	S (%)	I (%)	R (%)	MIC_50_	MIC_90_
Amoxicillin				4/4	>64/>64				2	>64				>64	>64				>64	>64
Amoxicillin- clavulanic acid (2:1)	94.4/94.4	0/0	5.6/5.6	4/4	8/8	94.8	0	5.2	2	4				>32	>32	90.0	0	10.0	2	8
Cefadroxil				8/8	16/16				16	16				>32	>32				8	>32
Cefalexin	94.7/94.4	0/0	5.3/5.6	8/8	8/8	93.5	0	6.5	16	16				>32	>32	60.0	0	40.0	8	>32
Cefovecin	92.6/93.2	2.8/2.5	4.6/4.3	1/1	2/1	93.5	0	6.5	0.5	0.5				>32	>32				1	>32
Cephalothin				8/8	16/16				4	8				>64	>64				4	>64
Gentamicin (* for cats)	97.5/98.8	0.4/0	2.1/1.2	1/1	1/1	81.8	2.6	15.6	0.5	8	88.9	11.1	0.0	2	4	70.0	0	30.0	0.5	>32
Neomycin				2/2	2/2				2	16				8	32				1	16
Enrofloxacin	88.0	2.8	9.2	0.03/0.03	0.25/0.5	1.3	71.4	27.3	0.25	>8	0	0	100	1	>8	53.3	0	46.7	0.06	>8
Marbofloxacin	89.4	1.4	9.2	0.03/0.03	0.25/0.5	71.4	1.3	27.3	0.12	4	0	22.2	77.8	0.5	2	53.3	0	46.7	0.06	>8
Orbifloxacin	91.2	1.8	7.0	0.12/0.12	0.5/2	42.9	29.9	27.2	2	>16				4	8	53.3	3.3	43.4	0.25	>16
Pradofloxacin	93.3	0.7	6.0	0.03/0.03	0.12/0.25				0.25	8				0.5	2				0.06	4
Trimethoprim- sulfamethoxazole (1:19) *	92.3/87.0	0/0	7.8/13.0	0.25/0.12	2/>8	76.6	0	23.4	0.25	>8				8	>8	66.7	0	33.3	0.5	>8

* Human derived clinical breakpoint. Data for dogs are indicated in black and data for cats are indicated in blue.

**Table 2:** Summary table with percentages of susceptible (S), intermediate (I), and resistant (R) Gram-negative UTI isolates from ComPath III from dogs and cats, together with the MIC50 and MIC90 values (µg/mL).

	*Escherichia coli* (Dog, n = 333/Cat, n = 183)					*Proteus* spp. (Dog, n = 101/Cat, n = 20)					*Pseudomonas* spp. (Dog, n = 22/Cat, n = 16)					*Klebsiella* spp. (Dog, n = 31)				
Antimicrobial Agent	S (%)	I (%)	R (%)	MIC_50_	MIC_90_	S (%)	I (%)	R (%)	MIC_50_	MIC_90_	S (%)	I (%)	R (%)	MIC_50_	MIC_90_	S (%)	I (%)	R (%)	MIC_50_	MIC_90_
Amoxicillin				4/4	>64/>64				1/2	>64/>64				>64/>64	>64/>64				>64	>64
Amoxicillin- clavulanic acid (2:1)	91.3/90.7	0/0	8.7/9.3	4/4	8/8	92.1/85.0	0/0	7.9/15.0	1/1	8/>32				>32/>32	>32/>32	93.5	0	6.5	2	8
Cefadroxil				8/8	16/16				16/16	32/>32				>32/>32	>32/>32				8	16
Cefalexin	94.9/92.9	0/0	5.1/7.1	8/8	16/16	83.2/65.0	0/0	16.8/35.0	16/16	32/>32				>32/>32	>32/>32	90.3	0	9.7	4	16
Cefovecin	94.3/93.4	1.2/0.6	4.5/6.0	1/1	2/2	98.0/85.0	0/0	2.0/15.0	0.25/0.25	0.5/>32				>32/>32	>32/>32				0.5	2
Cephalothin				8/8	32/32				4/4	16/>64				>64/>64	>64/>64				4	16
Doxycycline (* for cats)	0/86.9	1.2/3.8	98.8/9.3	2/1	16/8	0/10.0	0/0	100.0/90.0	64/32	>64/64				16/16	64/32	0	0	100	2	2
Gentamicin (* for cats)	98.8/98.9	0.3/0	0.9/1.1	0.5/0.5	1/1	90.1/90.0	2.0/0	7.9/10.0	1/0.5	2/4	90.9	9.1	0	2/2	4/2	93.6	3.2	3.2	0.25	0.5
Neomycin				1/1	2/2				2/2	32/8				8/8	32/16				1	1
Enrofloxacin	84.7	4.8	10.5	0.03/0.03	0.5/0.06	8.9	66.3	24.8	0.12/0.12	8/1	0	0	100	1/1	4/1	77.4	9.7	12.9	0.06	1
Marbofloxacin	86.8	1.8	11.4	0.03/0.03	0.5/0.06	79.2	0	20.8	0.06/0.03	2/0.25	0	18.2	81.8	0.5/0.5	2/1	87.1	0	12.9	0.03	1
Orbifloxacin	89.5	3.3	7.2	0.12/0.06	2/0.25	75.3	4.0	20.8	1/0.5	>16/8				4/4	16/8	87.1	3.2	9.7	0.12	4
Pradofloxacin	92.5	1.2	6.3	0.015/0.015	0.12/0.03				0.12/0.12	4/1				0.5/0.5	4/1				0.03	0.25
Trimethoprim- sulfamethoxazole (1:19) *	87.1/92.3	0/0	12.9/7.7	0.06/0.06	>16/0.5	79.2/75.0	0/0	20.8/25.0	0.12/0.12	>16/>16				8/8	>16/16	90.3	0	9.7	0.12	2

* Human derived clinical breakpoint. Data for dogs are indicated in black and data for cats are indicated in blue.

**Table 3:** Summary table with percentages of susceptible (S), intermediate (I), and resistant (R) Gram-positive UTI isolates from ComPath II from dogs and cats, together with the MIC_50_ and MIC_90_ values (µg/mL).

	*Staphylococcus intermedius* Group (Dog, n = 86/Cat, n = 20)					*Streptococcus* spp. (Dog, n = 53)					*Enterococcus* spp. (Dog, n = 49)/Cat, n = 36)					Coagulase-Negative Staphylococci (Cat, n = 38)				
Antimicrobial Agent	S (%)	I (%)	R (%)	MIC_50_	MIC_90_	S (%)	I (%)	R (%)	MIC_50_	MIC_90_	S (%)	I (%)	R (%)	MIC_50_	MIC_90_	S (%)	I (%)	R (%)	MIC_50_	MIC_90_
Amoxicillin				0.25/0.12	0.25/2				≤0.015	0.03				1/1	>64/1				0.12	0.5
Amoxicillin-clavulanic acid (2:1)	91.9/85	2.3/5.0	5.8/10.0	0.12/0.12	0.12/0.5				≤0.015	0.03	85.7/100	0/0	14.3/0	1/1	>32/1	89.5	10.5	0	0.12	0.5
Cefadroxil				1/1	2/4				≤0.25	≤0.25				>32/>32	>32/>32				1	4
Cefalexin				1/1	2/4				0.25	0.25				>32/>32	>32/>32				2	8
Cefovecin				0.25/0.12	0.25/1				≤0.03	≤0.03				>32/>32	>32/>32				0.25	2
Cephalothin				≤0.06/0.06	0.12/0.25				0.25	0.25				32/32	>64/64				≤0.06	0.25
Gentamicin *	88.4/85.0	2.3/5.0	9.3/10.0	0.12/0.12	8/8				32	32				16/16	>32/>32	97.4	0	2.6	≤0.06	0.25
Neomycin				≤0.25/8	16/16				128	>128				64/64	>128/>128				≤0.25	≤0.25
Enrofloxacin	44.2	45.3	10.5	0.12/0.12	0.5/>8	43.4	56.6	0	1	1				1/1	>8/>8				0.12	0.25
Marbofloxacin	20.9	67.5	11.6	0.25/0.25	0.5/>8	35.9	62.3	1.9	2	2				2/2	>8/>8				0.25	0.5
Orbifloxacin	90.7	0	9.3	0.5/0.5	1/>16	0	100.0	0	4	4				2/4	>16/>16				0.5	1
Pradofloxacin	90.7	1.2	8.1	0.03/0.03	0.06/2				0.12	0.12				0.5/0.5	2/2				0.06	0.25
Trimethoprim-sulfamethoxazole (1:19) *	88.4/80.0	0/0	11.6/20.0	0.25/0.5	8/8				0.06	0.12				0.03/0.06	>8/2	100	0	0	0.06	0.12

* Human derived clinical breakpoint. Data for dogs are indicated in black and data for cats are indicated in blue.

**Table 4:** Summary table with percentages of susceptible (S), intermediate (I), and resistant I Gram-positive UTI isolates from ComPath III from dogs and cats, together with the MIC_50_ and MIC_90_ values (µg/mL).

	*Staphylococcus intermedius* Group (Dog, n = 143/Cat, n = 15)					*Streptococcus* spp. (Dog, n = 64)					*Enterococcus* spp. (Dog, n = 62/Cat, n = 67)					Coagulase-Negative Staphylococci (Cat, n = 50)				
Antimicrobial Agent	S (%)	I (%)	R (%)	MIC_50_	MIC_90_	S (%)	I (%)	R (%)	MIC_50_	MIC_90_	S (%)	I (%)	R (%)	MIC_50_	MIC_90_	S (%)	I (%)	R (%)	MIC_50_	MIC_90_
Amoxicillin				0.5/0.5	16/64				≤0.015	≤0.015				1/1	4/2				0.12	4
Amoxicillin-clavulanic acid (2:1)	89.5/73.3	1.4/0	9.1/26.7	0.12/0.12	0.5/32				≤0.015	≤0.015	90.3/92.5	0/0	9.7/7.5	1/1	4/2	86.0	4.0	10.0	0.12	0.5
Cefadroxil				2/2	4/>32				0.12	0.12				>32/>32	>32/>32				2	4
Cefalexin				1/1	4/>32				0.25	0.25				>32/>32	>32/>32				1	8
Cefovecin				0.12/0.25	1/32				0.03	0.06				>32/>32	>32/>32				0.25	4
Cephalothin				0.06/0.06	0.25/64				0.12	0.12				32/32	>64/64				0.12	1
Doxycycline *				1/0.06	4/4	62.5	1.6	35.9	0.25	16	50.0/50.8	37.1/37.3	12.9/11.9	4/4	16/16				0.06	0.25
Gentamicin *	88.8/80.0	1.4/6.7	9.8/13.3	0.12/0.12	8/16				4	8				16/16	>32/>32	94.0	0	6.0	≤0.03	0.12
Neomycin				0.25/0.25	32/16				32	64				128/128	>128/>128				0.06	0.25
Enrofloxacin	40.5	43.4	16.1	0.12/0.12	2/16	48.4	51.6	0.0	1	1				1/1	>16/16				0.12	0.5
Marbofloxacin	18.2	62.2	19.6	0.25/0.25	4/16	78.1	20.3	1.6	1	2				2/2	>16/16				0.25	1
Orbifloxacin	86.0	1.4	12.6	0.5/0.5	16/>16	3.1	95.3	1.6	2	4				4/4	>16/>16				0.5	2
Pradofloxacin	89.5	3.5	7.0	0.03/0.03	0.5/2				0.12	0.12				0.25/0.25	8/2				0.03	0.25
Trimethoprim-sulfamethoxazole (1:19) *	85.3/80.0	0/0	14.7/20.0	0.5/0.5	8/16				0.06	0.06				0.03/0.06	16/>16	98.0	0	2.0	0.06	0.12

* Human derived clinical breakpoint. Data for dogs are indicated in black and data for cats are indicated in blue.

**Table 5:** Comparison between the proportion S, I, and R for *Staphylococcus intermedius* Group isolates against enrofloxacin and marbofloxacin based on CLSI Vet01S 6th and 7th Ed breakpoints.

	Breakpoints Ed6			Breakpoints Ed7		
	S	I	R	S	SDD *	R
**ComPath II**						
Enrofloxacin	90.7%	0%	9.3%	44.2%	45.3%	10.5%
Marbofloxacin	90.7%	0%	9.3%	20.1%	67.5%	11.6%
**ComPath III**						
Enrofloxacin	86.0%	4.9%	9.1%	40.5%	43.4%	16.1%
Marbofloxacin	86.0%	2.1%	11.9%	18.2%	62.2%	19.6%

* Susceptible-dose dependent.

**Table 6:** Breakpoints used for antimicrobials tested against bacteria isolated from cats (blue) and dogs (black) with urinary tract infection. When the same breakpoint was used for both dogs and cats, this is highlighted in green. Breakpoints in parenthesis are human derived.

	Breakpoints in µg/mL [Susceptible (≤)/Resistant (≥)] ^a^													
	Penicillins		Cephalosporins				Tetra- Cyclines	Aminoglycosides		Fluoroquinolones				Sulpho-Namides
	Amoxi-Cillin	Amoxicillin-Clavulanic Acid (2:1)	Cefa-Droxil	Cefa-Lexin	Cefo-Vecin	Cepha-Lothin	Doxy-Cycline ^e^	Genta-Micin	Neo-Mycin	Enro-Floxacin	Marbo-Floxacin	Orbi-Floxacin	Prado-Floxacin	Trimetho- Prim-Sulfa-Methoxazole (1:19)
*Staphylococcus* spp.	-	0.25/1	-	-	-	-	-	(4/16)	-	0.06/0.5	0.12/0.5	1/8	0.25/2 ^f^	(2/4)
*Streptococcus* spp.	-	0.25/1	-	-	-	-	(2/8) ^g^	-	-	0.5/4	1/4	1/8	-	-
*Enterococcus* spp.	-	8/16	-	-	-	-	(4/16)	-	-	-	-	-	-	-
*E. coli*	-	8/-	-	16/32; (16/32)	2/8	-	0.12/0.5; (4/16)	2/8; (2/8)	-	0.06/0.5	0.12/0.5	1/8	0.25/2	(2/4)
*Klebsiella* spp.	-	8/-	-	16/32; (16/32) ^c^	-	-	0.12/0.5; (4/16)	2/8; (2/8)	-	0.06/0.5	0.12/0.5	1/8	-	(2/4)
*Proteus* spp.	-	8/-	-	16/32; (16/32) ^b^	2/8 ^b^	-	0.12/0.5; (4/16)	2/8; (2/8)	-	0.06/0.5	0.12/0.5	1/8	-	(2/4)
*P. aeruginosa* ^d^	-	-	-	-	-	-		2/8	-	0.06/0.5	0.12/0.5	-	-	-
*Pasteurella* spp.	-	0.25/1	-	-	-	-			-	-	-	-	-	-

^a^ All breakpoints from CLSI document VET01S E7 [37]. ^b^ CLSI breakpoints for cefalexin and cefovecin are specific to *P. mirabilis* only. ^c^ CLSI breakpoints for cefalexin are specific to *K. pneumoniae* only. ^d^ CLSI breakpoints for *P. aeruginosa* are species specific. ^e^ Only tested in ComPath III. ^f^ Breakpoint only applicable to *S. intermedius* Group isolates. ^g^ Tetracycline clinical breakpoint. Organisms that are susceptible to tetracycline are also considered susceptible to doxycycline and minocycline. However, resistance to doxycycline and minocycline cannot be inferred from tetracycline resistance [37]. - no breakpoint available.

## Data Availability

The original contributions presented in this study are included in the article/Supplementary Material; further inquiries can be directed to the corresponding authors.

## Supplementary Materials

The following supporting information can be downloaded at: www.mdpi.com/xxx/s1-s8, Table S1: Activity of various antimicrobials against 284 *Escherichia coli* isolates cultured from dogs with urinary tract infections (Belgium, Czech Republic, France, Germany, Hungary, Italy, The Netherlands, Poland, Spain, Sweden, Switzerland, and United Kingdom isolates) (ComPath II); Table S2: Activity of various antimicrobials against 86 *Staphylococcus intermedius* Group isolates cultured from dogs with urinary tract infections (Belgium, Czech Republic, France, Germany, Hungary, Italy, The Netherlands, Poland, Spain, Sweden, and Switzerland isolates) (ComPath II); Table S3: Activity of various antimicrobials against 161 *Escherichia coli* isolates cultured from cats with urinary tract infections (Belgium, Czech Republic, France, Germany, Hungary, Italy, The Netherlands, Poland, Spain, Sweden, Switzerland, and United Kingdom isolates) (ComPath II); Table S4: Activity of various antimicrobials against 38 isolates of coagulase-negative staphylococci cultured from cats with urinary tract infections (Czech Republic, France, Germany, Italy, The Netherlands, Poland, Sweden, and Switzerland isolates) (ComPath II); Table S5: Activity of various antimicrobials against 333 *Escherichia coli* isolates cultured from dogs with urinary tract infections (Belgium, Czech Republic, France, Germany, Hungary, Italy, The Netherlands, Poland, Spain, Sweden, Switzerland, and United Kingdom isolates) (ComPath III); Table S6: Activity of various antimicrobials against 143 *Staphylococcus intermedius* Group isolates cultured from dogs with urinary tract infections (Belgium, Czech Republic, France, Germany, Hungary, Italy, The Netherlands, Poland, Spain, Sweden, and Switzerland isolates) (ComPath III); Table S7: Activity of various antimicrobials against 183 *Escherichia coli* isolates cultured from cats with urinary tract infections (Belgium, Czech Republic, France, Germany, Hungary, Italy, The Netherlands, Poland, Spain, Sweden, Switzerland, and United Kingdom isolates) (ComPath III); Table S8: Activity of various antimicrobials against 67 isolates of *Enterococcus* spp. cultured from cats with urinary tract infections (Belgium, Czech Republic, France, Germany, Hungary, Italy, The Netherlands, Poland, Spain, Sweden, Switzerland, and United Kingdom isolates) (ComPath III).
