# Supplementary material for: Antimicrobial Susceptibility of Canine and Feline Urinary Tract Infection Pathogens Isolated from Animals with Clinical Signs in European Veterinary Practices during the Period 2013–2018
Source: Antibiotics (Basel). 2024 May 28;13(6):500. doi: 10.3390/antibiotics13060500 (PMC11200364; doi:10.3390/antibiotics13060500)
Supplement: Supplementary file 1 [file antibiotics-13-00500-s001.zip › Table S1 Activity of various antimicrobials against 284 Escherichia coli isolates cultured from dogs with urinary tract infections.pdf]

Table S1. Activity of various antimicrobials against 284 *Escherichia coli* isolates cultured from dogs with urinary tract infections (Belgium, Czech Republic, France, Germany, Hungary, Italy, The Netherlands, Poland, Spain, Sweden, Switzerland, and United Kingdom isolates) (Compath II).

Antimicrobial Agent(s)	MIC values (μg/mL)																		MIC_{50} (μg/mL)	MIC_{90} (μg/mL)
	0.002	0.004	0.008	0.015	0.03	0.06	0.12	0.25	0.5	1	2	4	8	16	32	64	128	256		
Amoxicillin											25	167	40	2		2	48		4	> 64
Amoxicillin-clavulanic acid											43	172	53	11	5				4	8
Cefadroxil												4	230	34	3	13			8	16
Cefalexin												35	221	13	3	12			8	8
Cefovecin								5	90	135	33	8	2	1	3	7			1	2
Cephalothin											1	40	170	53	5	2	13		8	16
Gentamicin								3	90	182	2	1			2	4			1	1
Neomycin										42	232	5	1			2	2		2	2
Enrofloxacin			1	26	147	76	5	3	3	4	3		1	15					0.03	0.25
Marbofloxacin				18	191	41	4	4	4	5	1		4	12					0.03	0.25
Orbifloxacin				1	3	50	167	31	5	2	4	1	1	2	17				0.12	0.5
Pradofloxacin			7	113	118	17	3	7		2		5	7	5					0.03	0.12
Trimethoprim-sulfamethoxazole[a]					4	16	100	109	18	5	10	1		21					0.25	2

MIC_{50}: lowest concentration to inhibit 50% of bacteria; MIC_{90}: lowest concentration to inhibit 90% of bacteria.
The dilution ranges tested are those contained in the white area. Values shown above this range are greater than or equal to the concentration shown. Values at the lower end of these ranges are less than or equal to the lowest concentration tested. Where available, breakpoints are indicated by vertical lines.
[a] Indicates breakpoints derived from human breakpoints [37].
