# Supplementary material for: Antimicrobial Susceptibility of Canine and Feline Urinary Tract Infection Pathogens Isolated from Animals with Clinical Signs in European Veterinary Practices during the Period 2013–2018
Source: Antibiotics (Basel). 2024 May 28;13(6):500. doi: 10.3390/antibiotics13060500 (PMC11200364; doi:10.3390/antibiotics13060500)
Supplement: Supplementary file 1 [file antibiotics-13-00500-s001.zip › Table S5 Activity of various antimicrobials against 333 Escherichia coli isolates cultured from dogs with urinary tract infections.pdf]

Table S5. Activity of various antimicrobials against 333 *Escherichia coli* isolates cultured from dogs with urinary tract infections (Belgium, Czech Republic, France, Germany, Hungary, Italy, The Netherlands, Poland, Spain, Sweden, Switzerland, and United Kingdom isolates) (Compath III).

Antimicrobial Agent(s)	MIC values (µg/mL)																		MIC_{50} (µg/mL)	MIC_{90} (µg/mL)
	**0.002**	**0.004**	**0.008**	**0.015**	**0.03**	**0.06**	**0.12**	**0.25**	**0.5**	**1**	**2**	**4**	**8**	**16**	**32**	**64**	**128**	**256**		
Amoxicillin										1	19	150	83	3	1	3	73		4	> 64
Amoxicillin-clavulanic acid											28	199	77	14	9	6			4	8
Cefadroxil												13	263	36	7	14			8	16
Cefalexin												48	238	30	3	14			8	16
Cefovecin						1	1	20	111	159	22	4		1		14			1	2
Cephalothin											4	22	159	108	18	6	16		8	32
Doxycycline								4	32	119	119	13	11	13	8	10	4		2	16
Gentamicin								48	206	62	13	1		1		2			0.5	1
Neomycin								2	6	179	115	20	4		1	3	2	1	1	2
Enrofloxacin			3	87	170	22	4	12	7	6		1	3	6	12				0.03	0.5
Marbofloxacin			1	97	175	13	3	6	12	4		2	8	5	7				0.03	0.5
Orbifloxacin			1	9	23	127	114	10	4	10	9	2	2	1	21				0.12	2
Pradofloxacin		2	63	183	32	9	14	5	2	2	1	8	8	3	1				0.015	0.12
Trimethoprim-sulfamethoxazole[a]				2	41	168	56	14	5	3	1				43				0.06	> 16

MIC_{50}: lowest concentration to inhibit 50% of bacteria; MIC_{90}: lowest concentration to inhibit 90% of bacteria.
The dilution ranges tested are those contained in the white area. Values shown above this range are greater than or equal to the concentration shown. Values at the lower end of these ranges are less than or equal to the lowest concentration tested. Where available, breakpoints are indicated by vertical lines.
[a] Indicates breakpoints derived from human breakpoints [37].
