# Supplementary material for: Antimicrobial Susceptibility of Canine and Feline Urinary Tract Infection Pathogens Isolated from Animals with Clinical Signs in European Veterinary Practices during the Period 2013–2018
Source: Antibiotics (Basel). 2024 May 28;13(6):500. doi: 10.3390/antibiotics13060500 (PMC11200364; doi:10.3390/antibiotics13060500)
Supplement: Supplementary file 1 [file antibiotics-13-00500-s001.zip › Table S6 Activity of various antimicrobials against 143 Staphylococcus intermedius Group isolates cultured from dogs with urinary tract infections.pdf]

Table S6. Activity of various antimicrobials against 143 *Staphylococcus intermedius* Group isolates cultured from dogs with urinary tract infections (Belgium, Czech Republic, France, Germany, Hungary, Italy, The Netherlands, Poland, Spain, Sweden, and Switzerland isolates) (Compath III).

Antimicrobial Agent(s)	MIC values (μg/mL)																	MIC_{50} (μg/mL)	MIC_{90} (μg/mL)
	0.002	0.004	0.008	0.015	0.03	0.06	0.12	0.25	0.5	1	2	4	8	16	32	64	128		
Amoxicillin						23	5	17	29	35	11	5	3	1	4	9	1	0.5	16
Amoxicillin-clavulanic acid						22	75	31	2	6		3	1	2		1		0.12	0.5
Cefadroxil										10	118	4	3		1	7		2	4
Cefalexin								1	3	90	33	3	3	2	1	7		1	4
Cefovecin						19	55	52	2	1	1	3	1		1	8		0.12	1
Cephalothin					2	123	3	5	3	2	1			1	2		1	0.06	0.25
Doxycycline				1	22	38	5	2	2	2	2	58	10	1				1	4
Gentamicin[a]					3	21	83	17		1		2	2	9	3	2		0.12	8
Neomycin						8	18	48	15	3		3	6	22	14	5	1	0.25	32
Enrofloxacin				2	4	52	56	6	3	4	3	1	3	2	7			0.12	2
Marbofloxacin						2	24	89	8		3	3	2	4	8			0.25	4
Orbifloxacin					1	2	9	49	57	5		2	3	3	12			0.5	16
Pradofloxacin			5	30	76	12	1	4	3	2	7	3						0.03	0.5
Trimethoprim-sulfamethoxazole[a]						33	26	12	45	6		2	11	4	4			0.5	8

MIC_{50}: lowest concentration to inhibit 50% of bacteria; MIC_{90}: lowest concentration to inhibit 90% of bacteria.
The dilution ranges tested are those contained in the white area. Values shown above this range are greater than or equal to the concentration shown. Values at the lower end of these ranges are less than or equal to the lowest concentration tested. Where available, breakpoints are indicated by vertical lines.
[a] Indicates breakpoints derived from human breakpoints [37].
