# Supplementary material for: Antimicrobial Susceptibility of Canine and Feline Urinary Tract Infection Pathogens Isolated from Animals with Clinical Signs in European Veterinary Practices during the Period 2013–2018
Source: Antibiotics (Basel). 2024 May 28;13(6):500. doi: 10.3390/antibiotics13060500 (PMC11200364; doi:10.3390/antibiotics13060500)
Supplement: Supplementary file 1 [file antibiotics-13-00500-s001.zip › Table S7 Activity of various antimicrobials against 183 Escherichia coli isolates cultured from cats with urinary tract infections.pdf]

Table S7. Activity of various antimicrobials against 183 *Escherichia coli* isolates cultured from cats with urinary tract infections (Belgium, Czech Republic, France, Germany, Hungary, Italy, The Netherlands, Poland, Spain, Sweden, Switzerland, and United Kingdom isolates) (Compath III).

Antimicrobial Agent(s)	MIC values (µg/ml)																	MIC_{50} (µg/mL)	MIC_{90} (µg/mL)
	0.002	0.004	0.008	0.015	0.03	0.06	0.12	0.25	0.5	1	2	4	8	16	32	64	128		
Amoxicillin										5	12	106	23		1	3	33	4	> 64
Amoxicillin-clavulanic acid										5	18	116	27	9	4	4		4	8
Cefadroxil												8	143	18		14		8	16
Cefalexin[a]											1	49	103	17	1	12		8	16
Cefovecin						2		15	66	78	10	1	1			10		1	2
Cephalothin										1	8	17	95	39	8	2	13	8	32
Doxycycline[a]								2	26	93	36	2	7	7	6	4		1	8
Gentamicin[a]								21	121	33	5	1		1	1			0.5	1
Neomycin									8	92	68	11		1	2		1	1	2
Enrofloxacin			6	57	93	11	2	1	1	1			1	4	6			0.03	0.06
Marbofloxacin			1	64	95	7	2		2	1		1	4	3	3			0.03	0.06
Orbifloxacin				8	14	84	57	6		1	1	1		1	10			0.06	0.25
Pradofloxacin			44	106	17	2	1		2		1	4	4		2			0.015	0.03
Trimethoprim-sulfamethoxazole[a]				5	36	93	27	3	4	1			1		13			0.06	0.5

The dilution ranges tested are those contained in the white area. Values shown above this range are greater than or equal to the concentration shown. Values at the lower end of these ranges are less than or equal to the lowest concentration tested. Where available, breakpoints are indicated by vertical lines.

[a] Indicates breakpoints derived from human breakpoints [37].
